# Ambiguous landscapes: A framework for assessing robustness and uncertainties in archaeological point pattern analysis

**DOI:** 10.1371/journal.pone.0307743

**Published:** 2024-09-24

**Authors:** Eduardo Herrera Malatesta, Sébastien de Valeriola

**Affiliations:** 1 Centre for Urban Network Evolutions, Aarhus University, Aarhus, Midtjylland Region, Denmark; 2 QuaDiHum Lab, Université libre de Bruxelles, Brussels, Brussels Capital Region, Belgium; The University of Tulsa, UNITED STATES OF AMERICA

## Abstract

Landscape research in archaeology has greatly benefited from the increasing application of computational methods over the last decades. Spatial statistical methods such as point pattern analysis have been particularly revolutionary. Archaeologists have used point pattern analysis to explore spatial arrangements and relations between ‘points’ (e.g., locations of artefacts or archaeological sites). However, the results obtained from these techniques can be greatly affected by the uncertainty coming from the fragmentary nature of archaeological data, their irregular distribution in the landscape, and the working methods used to study them. Furthermore, the quantification of uncertainty in spatial data coming from non-systematic surveys has never been fully addressed. To overcome this challenge, archaeologists have increasingly relied on applying advanced methods from statistics, data science, and geography. While the application of advanced methods from formal sciences will provide robustness to models based on uncertain datasets, as with uncertainty, robustness must be assessed in relation to the case study, the regional context, and the methods used. These issues are of great importance when the models from advanced methods are directly used to create narratives about past landscapes. In this paper, we gather previous research on uncertainty quantification in archaeology and formalize its best practices into a framework to assess robustness and uncertainty in spatial statistical models, particularly focusing on one commonly used in the discipline, i.e., the Pair Correlation Function. This framework allows us to understand better how incomplete data affect a model, quantify the model uncertainties, and assess the robustness of the results achieved with spatial point processes.

## Introduction

This paper aims to contribute to the growing literature on data incompleteness and uncertainty quantification in archaeology by formalizing practices and procedures into a framework. With this, we aim to support colleagues who desire to better understand their models by quantifying their uncertainties. The framework we propose here focuses on assessing the robustness and uncertainties of the conclusions drawn from applying point pattern analysis to non-systematic regional data in archaeology. To achieve this aim, we have articulated the discussion on the reconstruction of past landscapes using computational methods around three key aspects: the use of point pattern analysis (PPA) in archaeology, the quantification of uncertainties, and the consideration of robustness. Point pattern analysis has several advantages and has represented an essential method in the archaeological toolset for the reconstruction of past landscapes. However, its applications have become increasingly popularized, and with it, there is the risk of overseeing the necessary critical reflection on the fundamentals and uncertainties of the methods used for point pattern analysis. To raise awareness of this, we will use as an example the widely used Pair Correlation Function (PCF) and Monte Carlo simulation. While we will use these methods to develop our framework, we will also present a critical assessment of their use, particularly in the context of databases created from non-systematic regional surveys.

The framework that will be presented is designed to aid archaeologists -working with datasets that are known to contain sources of uncertainty- to be able to apply spatial statistical methods and achieve a higher understanding of the uncertainties of the resulting models. We decided to develop and apply our framework to real archaeological data. While working with data simulated with the help of a theoretical model would be interesting, we decided to stay as close as possible to the actual practices of archaeologists.

The paper is structured to first present the case study and the methods used to analyze the archaeological data. Second, it explains the experiments designed to assess robustness and uncertainties. Third, it presents the results of the experiments. Finally, it discusses the advantages and disadvantages of the proposed framework.

### Archaeological landscapes

Landscape research in archaeology has greatly benefited from the increasing application of computational methods over the last decades. Spatial statistical methods such as point patterns have been particularly revolutionary [1, 2]. Archaeologists have used point pattern analysis to explore spatial arrangements and relations between ‘points’ (e.g., the locations of artifacts or archaeological sites). However, the results obtained from these techniques can be significantly affected by the uncertainty coming from the fragmentary nature of archaeological data, their irregular distribution in the landscape, and the working methods used to record and study them. To overcome this, since the 1960s, archaeologists have developed more systematic fieldwork methodologies, such as the total area survey strategy [3], as well as more explicit definitions of the archaeological site [4–7]. The systematic total area survey methodology proposed by Kowalewski [8] concerns the full coverage of a particular area or region. Yet, this methodology cannot be applied to every context as differences in topography and vegetation greatly impact survey strategies, and therefore, other non-systematic strategies must be followed [9]. To overcome the challenges posed by uncertain spatial data, there have been debates over the years about the advantages and disadvantages of using spatial statistics in archaeology [10, 11]. However, the quantification of uncertainty in spatial data coming from non-systematic surveys is still a field that needs more debate in archaeology, as well as the fundamental challenges of applying spatial statistical methods to study databases with partial evidence. In contexts where systematic total area survey is not possible, archaeologists have increasingly relied on applying advanced methods from statistics, data science, and geographical information systems to better understand and model the regional point patterns.

There is a growing trend of researchers working on improving data incompleteness and computational models [e.g., 11, 12–15]. Within this trend, the emphasis has been on studying archaeological patterns beyond the dataset. This means, to create experiments based on the observed or simulated data to better define the parameters and uncertainties in the methods used to understand past regional patterns. This opens up important questions, such as: what would my conclusions be if my data and/or model had been slightly different, for example, because they were collected/calibrated under different circumstances? Knowing that my data is inevitably incomplete and affected by my working methods, which models should I use to obtain the most solid conclusions? [16–21]. In this paper, we aim to contribute to this growing literature in archaeology by advancing the methodological challenges of quantifying uncertainties in regional point patterns.

### Point pattern analysis

**Fig 1:**
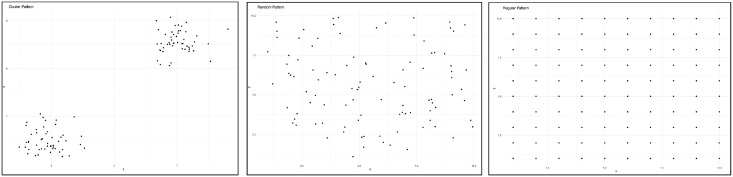
The three point patterns: Clustered (left), random (center), and regular (right).

Point Pattern Analysis (PPA) studies point patterns, i.e., the spatial arrangements of points in space. It has been used in archaeology since the beginning of regional archaeology in the 1950s [22, 23]. Its full potential concerning statistical analysis came with processual archaeology, particularly with the work of Hodder and Orton [1]. A vital aspect of studying spatial patterns is the notion of randomness. In order to determine the spatial structure of a pattern, all spatial patterns are contrasted against what is called Complete Spatial Randomness (CSR). For example, if an area is divided into quadrats and points are allocated to the area following a random uniform distribution, then every quadrat has an equal and independent chance of receiving a point, and every point has an equal and independent chance of being in any quadrat [1, 24]. Under the CSR assumption, the number of points in a given quadrat follows a Poisson distribution [25]. This theoretical random process can be used as a benchmark against which a particular pattern may be examined. When defining spatial patterns, there are three basic forms: points spaced regularly, scattered, and clustered (Fig 1). Clearly, the scale at which the pattern is defined is critical to fully understanding and representing a spatial pattern as a particular distribution may have different structures at different scales [26]. When doing PPA, the first task of the analyst is to define the type of spatial patterning to reject the presence of CSR. However, this step is just the beginning of exploring the point pattern, as identifying the pattern does not mean that anything has been explained. The identification is simply an aid in the interpretation of the spatial process that produced the pattern [25]. There are several methods to test for CSR, for example, the quadrat method. However, as archaeological distributions usually contain a small number of points, other statistical tests, such as the Kolmogorov-Smirnov (K-S) test and Clarke and Evans *R* statistic, have been more common [27, 28]. These are statistical tests for assessing the distribution and spatial randomness of data, yet they differ in that the K-S null hypothesis is that the sample comes from the same distribution for a two-sample test or that the sample comes from a specified reference distribution for a one-sample test. The *R* statistic null hypothesis is that the points are randomly distributed within the given area, indicating spatial randomness. Still, considering the complexity of the archaeological record, more advanced methods, such as the *K* and *L* functions or the Pair Correlation Function, have been preferred and successfully used to assess the CSR hypothesis and obtain a deeper understanding of the point pattern [2, 29–32].

### Uncertainty in archaeology

Uncertainty is a part of our degree of knowledge. As such, its scope and sources must be explicitly defined to provide a secure basis for treating archaeological data, analyses, and final narratives. The formal study of uncertainty, defined as uncertainty quantification (UQ), has been mainly developed in the fields of computer science and mathematics [33, 34]. However, there are interesting contributions from other fields ranging from engineering to model-based management [35, 36]. The application of concepts defined and tools developed in this discipline has been increasing in the last decade in the fields of archaeology [e.g., 37–41] and the digital humanities [e.g., 42–46].

UQ researchers have proposed categorizing uncertainty into two broad types: aleatoric and epistemic [47]. On the one hand, aleatoric uncertainty represents the intrinsic randomness of the phenomenon under study. To explore this type, mainly two families of methods have been used in computational analyses to study spatial analysis models: spectral [48] and simulation-based [49]. A simplistic archaeological example of this uncertainty type would be the shape variations of some tools. For example, two obsidian blades will always have slightly different shapes, even if manufactured using the same process. This type of uncertainty is not a matter of primary importance in archaeology or, more broadly, in the humanities. On the other hand, epistemic uncertainty is the result of the imperfection of our knowledge. It can be quantified using diverse methods, particularly techniques using random processes to cope with the part of reality we do not know [50]. Continuing with the example of obsidian blades, even if we ignore from which quarry the stone used to make the blades come from, epistemic uncertainty may be reduced by expanding the research and its empirical efforts. A research project exploring the quarries around the site where the blades were found would answer the question of their provenance. This second type of uncertainty is of great significance in archaeology and the humanities.

Epistemic uncertainty can originate from both the data and the models. Several sources of uncertainty can be identified when analyzing spatial data from non-systematic regional surveys in archaeology. Some key examples are, first, the field survey uncertainty, particularly regarding recording archaeological evidence. This refers to issues of transparency of the methodology used, the reproducibility of the method in similar contexts or by other researchers, and the accuracy of the tools and technologies used to record the surface or stratigraphical evidence. Second, the uncertainty related to the definition of archaeological sites. While several decades ago, there was a lot of debate on how to classify archaeological sites [4, 6, 51–53], that debate seemed to have been somehow neglected by most researchers, and explicit accounts of site classification are rarely found in contemporary archaeological literature. Third, there is uncertainty embedded in the databases. This has to do with the process and software researchers use to create and secure a database over time, as well as the decisions taken when combining new and legacy data [54]. Fourth, there is uncertainty in the visualization tools and methods we use to represent data [55]. What does a “dot” mean on a map? How do we differentiate different types of sites for analysis? Archaeological literature often uses distributional maps to represent spatial data. Yet, little to no information is often provided on the methodologies used to classify or categorize those “dots” or if they are all equally important to be represented in the same way [e.g., 45].

Three families of methods have been used to study the fragmentary nature of archaeological data in the context of landscape and computational archaeology: probabilistic models such as Monte Carlo simulation [e.g., 18, 37, 56], Bayesian approaches [e.g., 57–59], and Sensitivity Analysis [e.g., 60, 61]. These methods provide quantitative accounts of the variance in the datasets and model results as well as further information about their internal structure. This paper will contribute to this growing literature on uncertainty quantification in archaeology by studying contexts where a high degree of spatial uncertainty might affect past landscape reconstructions, such as databases created from non-systematic regional surveys.

### The concept of robustness

In statistics, robustness has been defined as the “insensitivity [of the results] to small deviations from the assumptions” [62]. A procedure is robust if it leads to a conclusion that is “supported by strong data-based evidence and not simply by a discovery gleaned from a preconceived model and weakly supported by a part of the data” [63]. Robust tools have “levels of performance that are consistently high for processes that obey realistic deviations from the model, i.e., for processes in the neighborhood of the model” [64]. However, there have been extensive debates regarding the definition of robustness and the optimal procedures to achieve it [see e.g., 64–66].

How do these definitions transpose to archaeology and, more specifically, to archaeological studies based on non-systematic regional surveys? The “assumptions” of Huber’s definition correspond here to the properties of the data and the working methods used to formulate archaeological conclusions. As we saw in the previous section, these are subject to uncertainties, i.e., they can be considered variables in the term’s primary sense. The difference between what is observed and what could have been observed (if the real-world process that led to the data had gone differently) constitutes the “small deviations” of the definition. “Insensitivity” is to be understood here from a global perspective, that of the research narrative. In other words, what is important here is that the conclusions of the archaeological analysis are not drastically affected or change when the uncertain elements used to reach those conclusions vary. To summarize, a robust tool leads to archaeological conclusions that are minimally affected by the deviations induced by the uncertainty types we identified above.

In archaeology, the search for robustness is usually based on the question of how to achieve significant models when you do not trust your data. While advanced computational methods are useful to bring robustness to archaeological models, they should not be used uncritically, as due to the fragmentary condition of the archaeological record, all datasets are uncertain, and that uncertainty can filter towards the model result if not explicitly considered in the analytical process [e.g., 67–69]. Therefore, one could ask, how robust is a model based on uncertain data, even if the model itself can be considered robust? This could lead to the definition of a robust procedure as one that starts with the assumption that the data is uncertain and biased. Then, the model will probably deviate from a hypothetical assumption of what will be the scenario if the data is complete and/or less uncertain. Here, we understand ‘scenario’ as the result of a model. It is not, thus, about the uncritical application of advanced statistical or mathematical models but about creating a transparent and replicable procedure. In this sense, robust approaches in archaeology, and more broadly in the humanities, seek to develop experiments to test the performance of the data and the model against a large number of simulations that will produce a variability of scenarios [e.g., 70–72]. In this paper, we refer to these last scenarios as ‘robustness scenarios.’

## Material and methods

This section will first present the archaeological context of the case study. Then, we will discuss the uncertainties embedded in the Monte Carlo simulation and how they affect the models. Finally, we will focus on the methodological aspects of the uncertainty and on the robustness framework we propose.

### Case study

**Fig 2:**
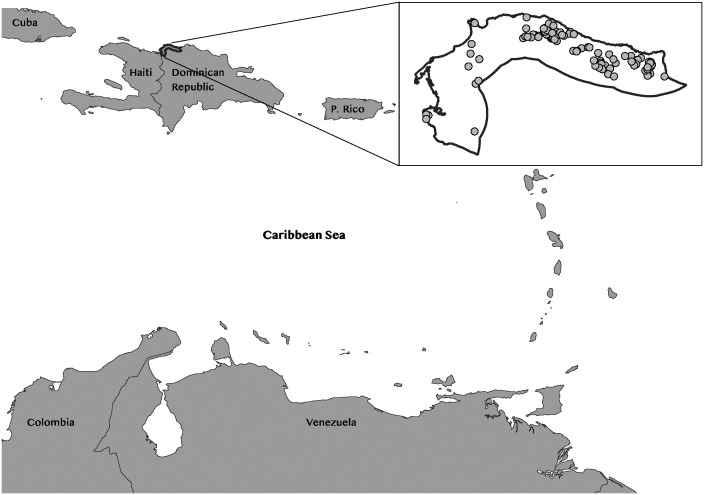
The research area and sites in the Caribbean context (The base map was created using the Natural Earth public domain data, scale 1:10m).

This paper applies the uncertainty and robustness assessing framework described in the following section to a point pattern analysis of Indigenous sites in the northwestern Dominican Republic. More precisely, we consider a dataset from a non-systematic regional survey on a 10km-wide band along the coast of the Montecristi coast [73, 74, Fig 2]. This database consists of 102 archaeological sites and is available open access [75]. As a result of the complex topography and vegetation of the area, the surveys were done in an opportunistic manner [24]. This means the places with archaeological materials were located based on the knowledge of different people from various communities of the Montecristi province.

The dataset combines habitation sites and exploitation of sea resource areas. All sites were defined by the presence of material culture (ceramics, lithics, shells) on the ground’s surface, and each site’s functionality was determined by the type of materials and their combination. The habitation sites were defined by their location away from the coastal line, mostly in the mountains and valleys, the presence of concentrations of ceramics, lithics, and shell objects, as well as, in some sites, the presence of artificial earth mounds and platforms. The concentrations, combinations, and spread over the terrain of the material culture vary among sites, which led to the definition of three size categories (small, medium, and large). The exploitation of sea resource areas were defined by their location on or close to the coastal line, the presence of a large number of shells, mostly (*Lobatus gigas*), and a small presence or complete absence of ceramics. The exploitation of sea resources type was defined as areas and not sites, as their distribution on the surface ran for several kilometers, and it was complicated to define where one area finished and the other star. This complicated the transformation of areas into points that could be used for spatial point pattern analysis, and therefore, in the original research and in this paper, these areas are not taken into account for spatial analysis.

The archaeological sites were classified following two stages in the original research and fieldwork [73]. The first stage was during fieldwork and consisted of identifying any given number of material culture in the ground as independent manifestations and grouping them into three categories: cluster, dispersed, and isolated finds. These groups were defined as *spatial datasets*. During the fieldwork, an individual code was assigned to each of these spatial datasets (e.g., “MC-1”, where “MC” corresponds to Montecristi and the number to the group of materials recorded in increasing order). A total of 673 spatial datasets were recorded within the study area. The second stage was made after fieldwork and was based on statistical analysis. For this, a Clarke and Evans [28] Nearest Neighbor analysis (NN) and a Histogram of Nearest Neighbor (HNN) were performed to assess at which distances the distribution of spatial datasets was clustered. The results of the NN showed a highly clustered pattern, and the HNN exposed that the distances between points were no greater than 100 m. Based on this analytical process, a *site* was defined in that research as “the spatial clustering of material culture, which can be observed in the form of clusters, dispersions, and/or isolated findings, which do not have more than 100 m of separation between each evidence” [73]. Due to the characteristics of the exploitation of sea resource sites, we decided for this paper to only use the habitation ones, totaling 95 sites. For the analysis, we use the information regarding the geographical location and size of the sites. All codes and analyses were performed using the statistical software R, version 4.3.1 [76]. For the specific analysis, the packages spatstat [77], rgdal [78], maptools [79, 80], tidyverse [81, 82], gridExtra [83], scales [84], and ggspatial [85] were used. All codes can be found in the supporting information at the end of this paper.

### Revising Monte Carlo simulation in archaeology

Monte Carlo statistical methods have been applied in archaeology for over two decades to explore statistically significant spatiotemporal patterns and model archaeological uncertainties [e.g., 59, 86–89]. While its applications have greatly improved archaeological models and explanations of the past, a reflection on how to adjust this method to specific archaeological data and contexts has been lacking. One specific point that deserves attention and more discussion in the literature is the decisions behind the selection of the number of simulations used to generate the Monte Carlo envelope. This simulation envelope aims to assess the null hypothesis of CSR, i.e., to assess if the observed point distribution is significantly different from a Poisson process [90, 91].

Most of the literature that provides descriptive explanations of the use of the method follows a standard 999 or 1,000 iterations to create the simulation envelope [e.g., 11, 30–32, 37, 92]. Yet, besides referencing works from other fields, no explanation is given for the reason for using this number of iterations and how that relates to the specific archaeological dataset used. In archaeology, some exceptions of research that have provided explanations on the use of a different number of scenarios than the standard in archaeology are the work of Crema and Kobayashi [93], who used 5,000 scenarios for their simulation envelope, and DiNapoli and colleagues [94], who used 39 simulations (in a more complex context). Baddeley and colleagues [90] indicate that the number of scenarios should respond to the specific case under study; this is a consideration of the sample size and the significance level at which the analyst is satisfied with rejecting the null hypothesis. In archaeology, the standard and accepted confidence level used to assess the null hypothesis is generally considered to be 95% [see e.g., 27]. However, databases with known levels of uncertainty and bias should use a higher significance level for the rejection of the null hypothesis. In this paper, to provide a wide variety of situations, we work with confidence levels of 90%, 95%, 98%, 99% and 99.5% (see the Discussion section).

The lack of explanations on the decision to use a specific number of simulations might become a problem when a method starts being popularized, and parameters are being used with no real consideration of the underlying reasons for its use. While our objective is not to criticize previous work, the point we aim to raise here is to highlight that, during the development of this paper, we realized that the number of iterations for the Monte Carlo simulation envelope in cases where there is a high degree of uncertainty is essential to better understand its role in the model as well as to obtain robust results. We recommend that because of these aspects, an explicit revision of the way we use this method in archaeology should be done. With this paper, we want to contribute to such revision.

### Robustness assessing framework

This section will present our robustness assessing framework. This framework is a formalization of an approach that has been previously used in computational archaeology and network science [e.g., 70, 71]. The main idea of this framework is to generate *n* (a relatively large number of) randomly simulated scenarios (that we will call the *robustness scenarios*) representing alternative states of reality. These alternative states of reality correspond to what might have happened if conditions (e.g., decision during field survey, accessibility to sites, recoding of a different number of sites, etc.) had been slightly different, i.e., if the uncertain elements against which robustness is tested had taken on different values from those observed. Then, we compare what is observed in each robustness scenario with the actual data (i.e., what is observed in reality). The differences between the two are the “small deviations from the assumptions” referred to in the definition of robustness (now simply called *deviations*). Assessing robustness consists of quantifying these differences.

This can be seen as a Monte Carlo framework since it involves generating scenarios that mimic the possible values of some uncertain elements or, in other words, creating (based on some probability distribution, such as a uniform or normal distribution) repeated random samples (a model of possible outcomes), to compute numerical quantities [95]. Note that the robustness scenarios we create in the robustness assessing framework are not the same as the Monte Carlo simulation envelopes used to construct the CSR quantile envelope.

The robustness assessment framework presented in this paper has three building blocks: the observable, the experiment, and the comparison tool. This can be seen as a Monte Carlo framework since it involves generating scenarios that mimic the possible values of some uncertain elements [91]. The observable, a term borrowed from quantum mechanics which refers to any measurable entity [96], has to do with the original results of the PCF applied to the Montecristi data. The experiments deal with the process by which we replicate the deviations. The comparison tool measures the impact of deviations on *V* (see the below section).

As with any Monte Carlo approach, the number of robustness scenarios of our framework is a crucial parameter to determine. After testing several values, we decided to use *n* = 10,000. This choice is justified in the "Results" section.

#### The observable: A point clustering metric

The first building block of the framework is the quantity whose changes we measure when considering deviations. We will call this the *observable* and denote it by *V*. Since the aim is to test the robustness of archaeological conclusions and, by that, assess uncertainty, *V* must be at the heart of the research narrative, i.e., our argument must contain a sentence like “as the value of *V* is …, we conclude that…”. It is the value we actually compute from the original dataset, *V*(*observed*), that we will compare with the values it takes in each of the randomly simulated scenarios, *V*(*scenario*_*i*_), for *i* = 1, …, *n*.

In a previous archaeological analysis of this data, Herrera Malatesta [7] observed that the archaeological site distribution in this area consisted of a clustered pattern at small-scale distances and a dispersed pattern at large scales. In that study, interpreting the clustered pattern was essential for creating narratives about the past Indigenous people. Based on this, in this paper, we are interested in assessing the robustness and uncertainties of this clustering, on the one hand, to challenge the previous results and, on the other, to have a point of reference for the results in this new study. To do this, we chose the Pair Correlation Function (PCF) metric, also used in the original work [7], to estimate the spatial pattern of the area. The PCF, or radial distribution, has been regarded as the most important tool for the analysis of spatial point patterns [97]. It is defined as “the probability of observing a pair of points of the process separated by a distance *r*, divided by the corresponding probability for a Poisson process” [25]. This method provides an estimation of the degree of spatial clustering and regularity of the spatial pattern at multiple scales within a window of observations. This allows a better differentiation of patterns at different scales [25, 97].

For this paper, we used the inhomogeneous function based on an inhomogeneous Poisson process, which assumes that the underlying intensity is not constant but varies across the study area. As established in previous publications [32], we know this is an inhomogeneous pattern. To address edge effects, we used Ripley’s isotropic correction, an edge correction method, to calculate the summary statistic. Edge correction methods are techniques used for eliminating the bias generated by the edge effects [25].

#### The experiment: Sampling of archaeological sites

The second building block of the framework is the process by which we replicate the deviations that we call the *experiment*. It is a way of generating random scenarios that take the observed situation and modify specific parameters to obtain alternative states of reality. The experiment is closely linked to the type of uncertainty being tested, as the generated scenarios should explore all the possibilities it opens.

As mentioned, archaeological materials are samples of the entire cultural repertoire the people used and produced in the past. From this sample, a database is created. In cases where the collection strategies used in the field do not apply random sampling or a systematic total area survey, the data creation may contain high levels of uncertainty. Then, how can we be sure that the database used for computational analysis is a representative sample of past realities? A solution to bring robustness to the analysis and, at the same time, quantify the uncertainties in the model results is to create experiments that extract systematic amounts of data from the original dataset and a large number of simulations are calculated to test the data and the models.

The main idea of the present study is to assess the robustness concerning the incompleteness of the database, or in other words, the fact that some sites are missing from our records. To simulate this situation, we start from our database and generate scenarios in which some sites have been randomly removed to compute a Pair Correlation Function (PCF) in each scenario and compare the obtained value with the original results. More precisely, we deduct 10%, 20%, 30%, 40%, and 50% of the database (*sites* = 95) to observe how much these removals impact the results. To do so, we must define a way to sample the sites to be removed, that is, a probability distribution. We have considered two different probability distributions, which correspond to each of our two experiments.

In the first experiment (E1), we use a uniform distribution. This means that all sites have the same chance of being picked. This is the most straightforward choice but far from the archaeological fieldwork’s reality. In the second experiment (E2), we introduce some inhomogeneity by deciding that large sites are more likely to be discovered than small ones. More formally, we define the probability that a given site is kept in the database (i.e., the probability that it is not removed) as proportional to its area. This way, we account for very large sites being more prominent and unlikely to be unregistered during fieldwork.

Finally, we should note that each portion of data removed (10% to 50%) leads to a different final number of data points. Therefore, as previously explained, we needed to build a different CSR envelope for each of these cases and generate each time a set of Monte Carlo simulations (using exactly the same process as for the generation of the CSR envelope for the whole dataset, except that here we work with fewer points). For the calculations of these simulations, the resources of a computing cluster were used.

#### The comparison tools: Frequencies and interval midpoint densities

The third building block of the robustness framework is a way of measuring the impact of deviations on *V*, the *comparison tool*. Once the *n* scenarios have been generated, we must compare *V*(*observed*) to *V*(*scenario*_*i*_) in each of them.

We will look at two comparison tools. The first one is the frequency at which (i.e., the percentage of scenarios in which) the first-level conclusion of the point cluster analysis is the same as with the original data. That first-level conclusion is “the sites are clustered” or, in other words, “we observe a significant difference with respect to the CSR Poisson null model.” To do that, we create a binary indicator for each robustness scenario indicating if, in that scenario, the PCF curve rises above the corresponding CSR envelope quantile. Then, we average these binary indicators over all the robustness scenarios to obtain a frequency.

For the second comparison tool, we look at second-level conclusions that can be drawn from the clustering analysis, i.e., the further information we can get from the PCF when we already know that the sites are clustered. We have chosen to look at the interval of distances at which the observed PCF curve rises above the CSR envelope quantile. In other words, we now consider the distance values between pairs of points in the dataset that are sur-represented concerning the CSR Poisson null model. This gives information about the “typical distance” between the considered sites. To do this, we filter the robustness scenarios to retain only those with a clustering of sites (corresponding to a binary indicator equal to 1, as defined in the previous paragraph). We then calculate the midpoint of the corresponding distance interval (of the first interval, when there is more than one) in each scenario. We then plot density curves for these interval midpoints to compare their distribution. These plots allow us to observe where the sites tend to be located on the inter-site distance scale in relation to each other. From here, the results will be described based on the plots.

## Results

In order to present a contrast between the traditional way in which PCF and Monte Carlo simulation envelopes are used in archaeology and the framework we are suggesting in this paper, the results are divided into two groups: the intuitive and the quantitative results. The first one is used to interpret the PCF models corresponding to the ‘original results’ and the ‘experiments.’ The second one is represented in the comparison tool. In addition, and to further our previous discussion on the use of the Monte Carlo envelope in archaeology, in the section about the ‘original results’, we present the variability of what can result from the use of the Monte Carlo simulation envelope.

### The original results

**Fig 3:**
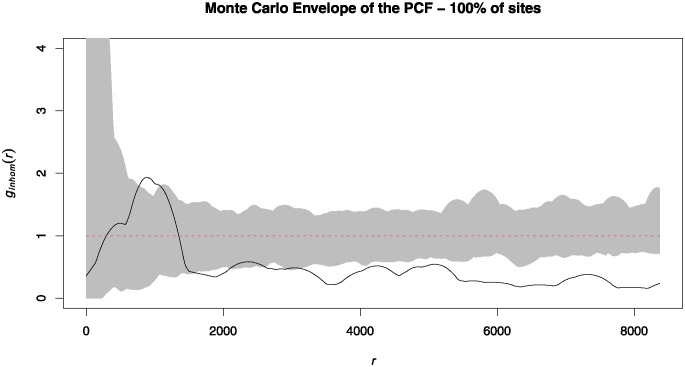
Pair Correlation Function (PCF) of the original database (*sites* = 95), with a Monte Carlo envelope at 1,000 simulations.

When performing a PCF with a 1,000 simulation Monte Carlo envelope on the entire database, the resulting model indicates a cluster pattern between ~250 to 1,500 m, with significant values at ~1,000 to 1,250 m. Then, the pattern becomes regular, having significant values at several distances after ~1,800 m (Fig 3). In this case, the statistically significant areas are considerably affected by the extreme scenarios produced by using only 1,000 simulations.

### The original results with modified Monte Carlo envelopes

**Fig 4:**
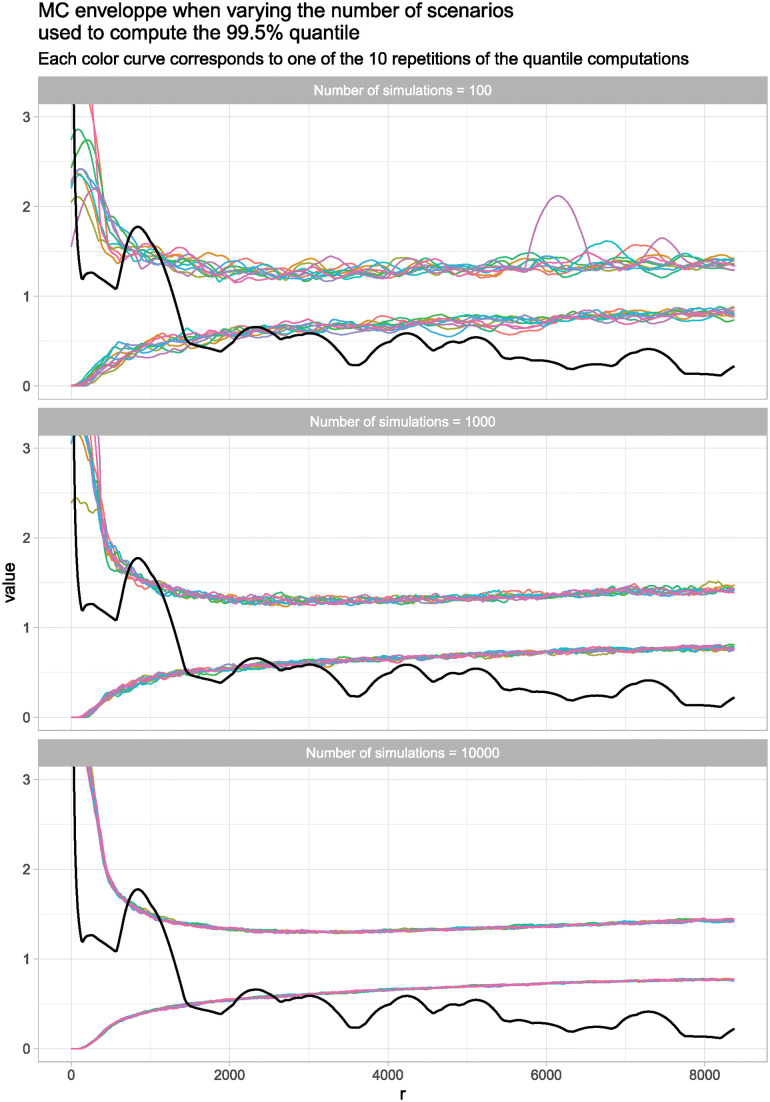
Pair Correlation Function (PCF) of the original database (*sites* = 95) with a Monte Carlo envelope of 100, 1,000, and 10,000 simulations.

**Fig 5:**
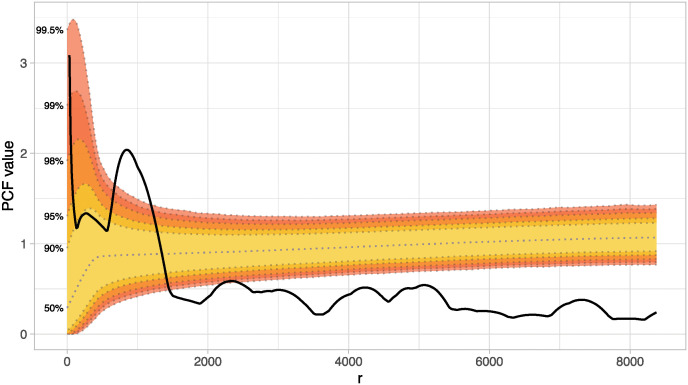
Pair Correlation Function (PCF) of the original database (*sites* = 95) with a Monte Carlo envelope at 100,000 simulations.

To show the variability of the model outcome, we compute the Monte Carlo envelope with varying numbers of scenarios of 100, 1,000, and 10,000 simulations, using the 99.5% quantile level (Fig 4). In each case, we repeat this calculation ten times, each time with a different random seed, to show how the resulting quantiles change from one time to the next. The reason we did not do this calculation for 100,000 simulations is that such a 10-fold repetition is beyond the reach of our computing resources. The various results show the impact that different numbers of simulations can have on the overall pattern and, therefore, how it can impact its interpretation. With 100 simulations, there is no significant cluster pattern in each batch of simulations, while with the 1,000 and 10,000 simulation Monte Carlo envelopes, the results reflect with varying degrees of fidelity the original envelope results and maintain a similar spatial structure. As we still observe some slight variations from one curve to another using 10,000 simulations, we have opted, in the following analyses, for 100,000 simulations. In this way, the patterns become quite clear (Fig 5).

### Visualizing individual robustness scenarios

**Fig 6:**
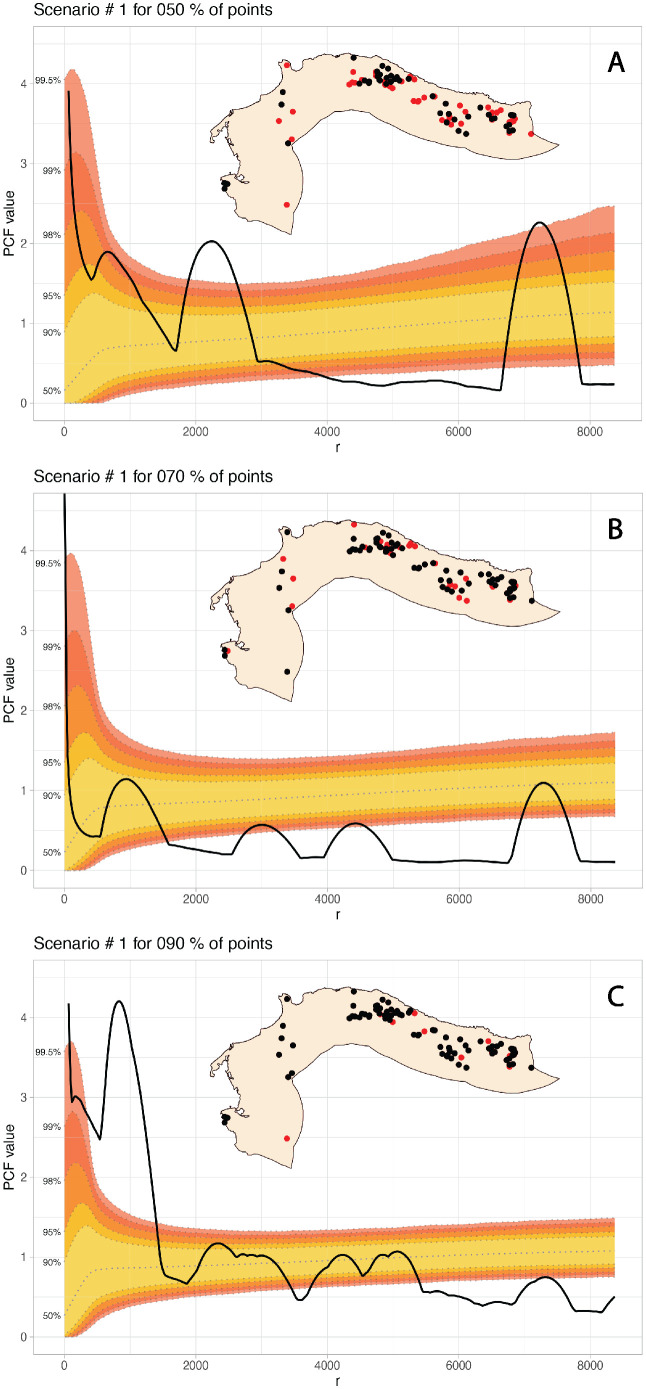
PCF of the E1 at different sampling percentages with the 100,000 simulations MC envelope. The red dots represent the removed sites in each scenario.

The first experiment considers the calculation of a Pair Correlation Function with a Monte Carlo envelope composed of 100,000 simulations on five groups of sample data. These groups were created by random sampling archaeological sites from the original database at 10%, 20%, 30%, 40%, and 50%. As expected, by removing a larger sample of sites from the database, the envelope got wider as the scenarios for what can be expected under a random distribution are more diverse. Also, by removing more sites, the observed point distribution gets a different pattern in each scenario. Fig 6 shows an example of the first scenario for the extraction of 10%, 30%, and 50% of sites from the database. We visually explored 50 of the 10,000 of these robustness scenarios, which can be accessed in the supporting information.

**Fig 7:**
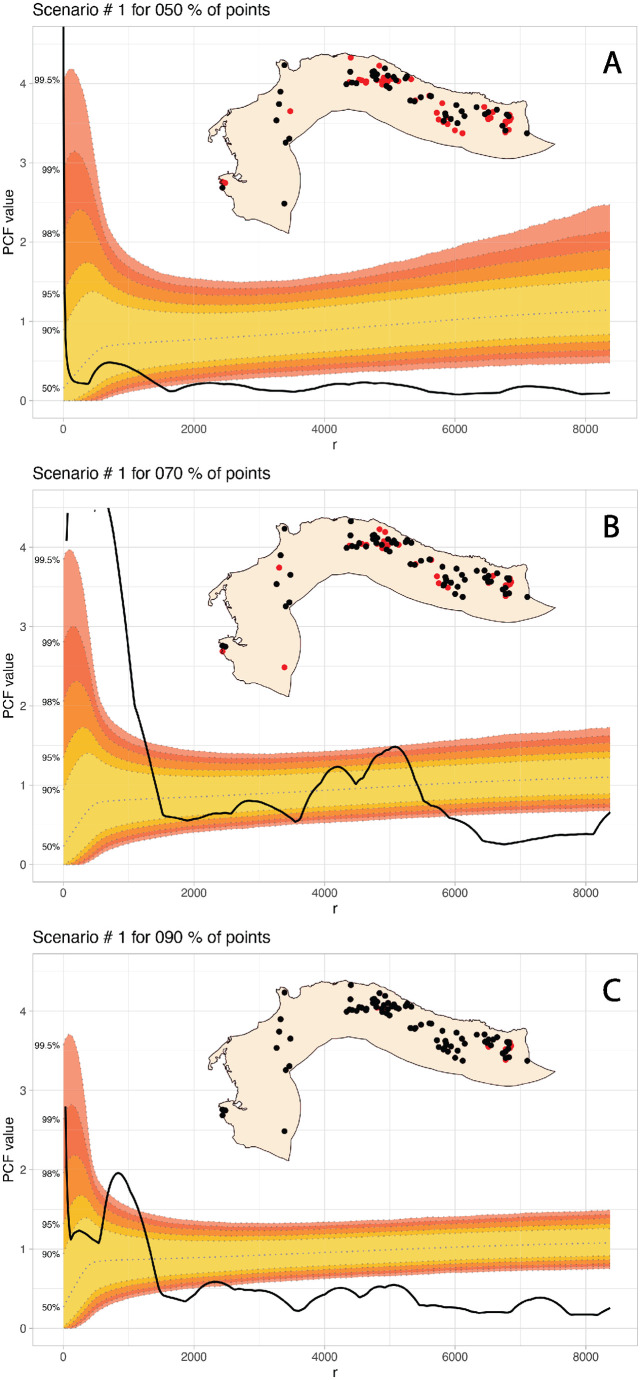
PCF of the E2 at different sampling percentages with the 100,000 simulations MC envelope. The red dots represent the removed sites in each scenario.

For the second experiment, we aimed at adjusting the sampling method so that the selection of archaeological sites is weighted to increase the chances for the small sites to be left out. This is because the medium and large sites were easier to record in the field than the small ones, which were harder to spot on the ground. As in the previous case, a simple visual assessment indicates that both the observed point distribution and the Monte Carlo envelope are more affected when removing a large sample of sites (Fig 7).

### The comparison tools

**Fig 8:**
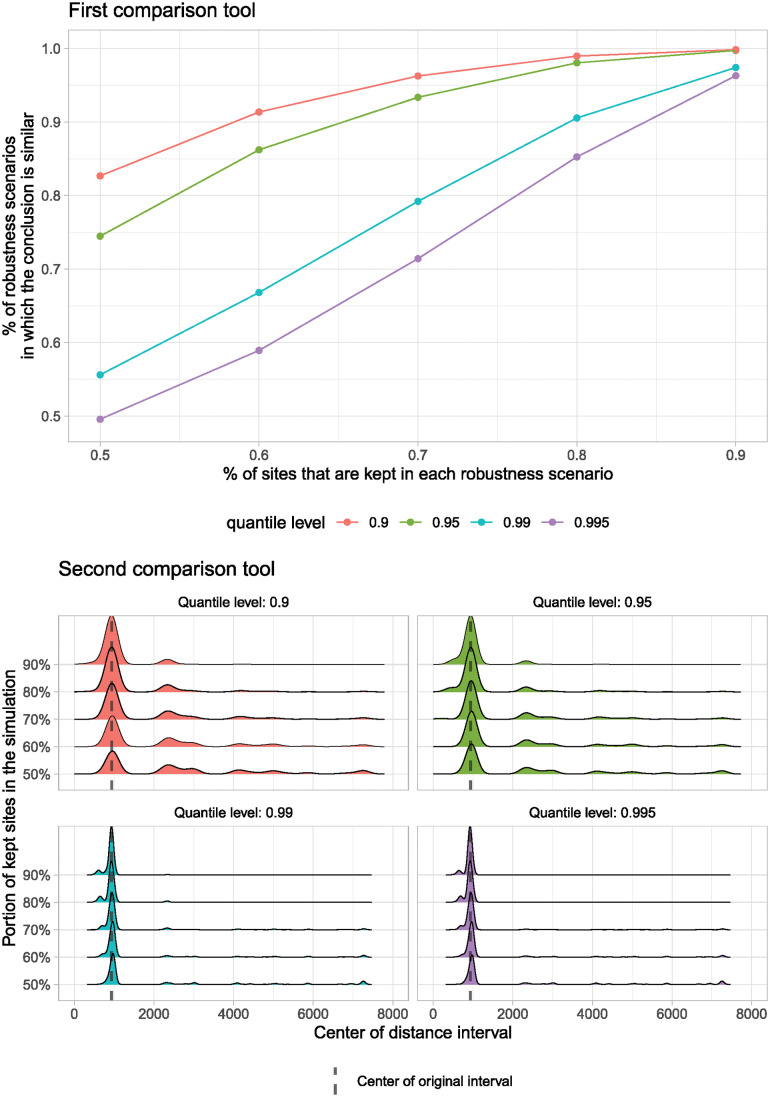
The comparison tools for the first experiment (E1).

The results from applying the two comparison tools allowed a straightforward assessment of which experiment and sampling strategy provided the most robust results and the associated uncertainties. Regarding the first comparison tool (Figs 8 top and 9 top), it is possible to observe that there are differences in the frequencies at which we reach the same first-level conclusion. For the E1, the pattern shows that when removing 50% of the database at random, there is a threshold between 50% and ~85% (depending on the chosen quantile level) in which the conclusions will remain the same as with the original model based on a 100,000 simulation Monte Carlo envelope. At 70% of the database, the threshold of robustness scenarios where the conclusion remains the same, increases to ~70% and ~95%. This means that when removing 30% of the data, at the level of the 0.995 quantile, there is a ~30% uncertainty that the model results will produce different scenarios with respect to the original model. Whereas for the 0.9 quantile there is a 5% of having an alternative result. As expected, as less data is removed from the original database, the percentage of robustness scenarios increases, and the uncertainty decreases.

**Fig 9:**
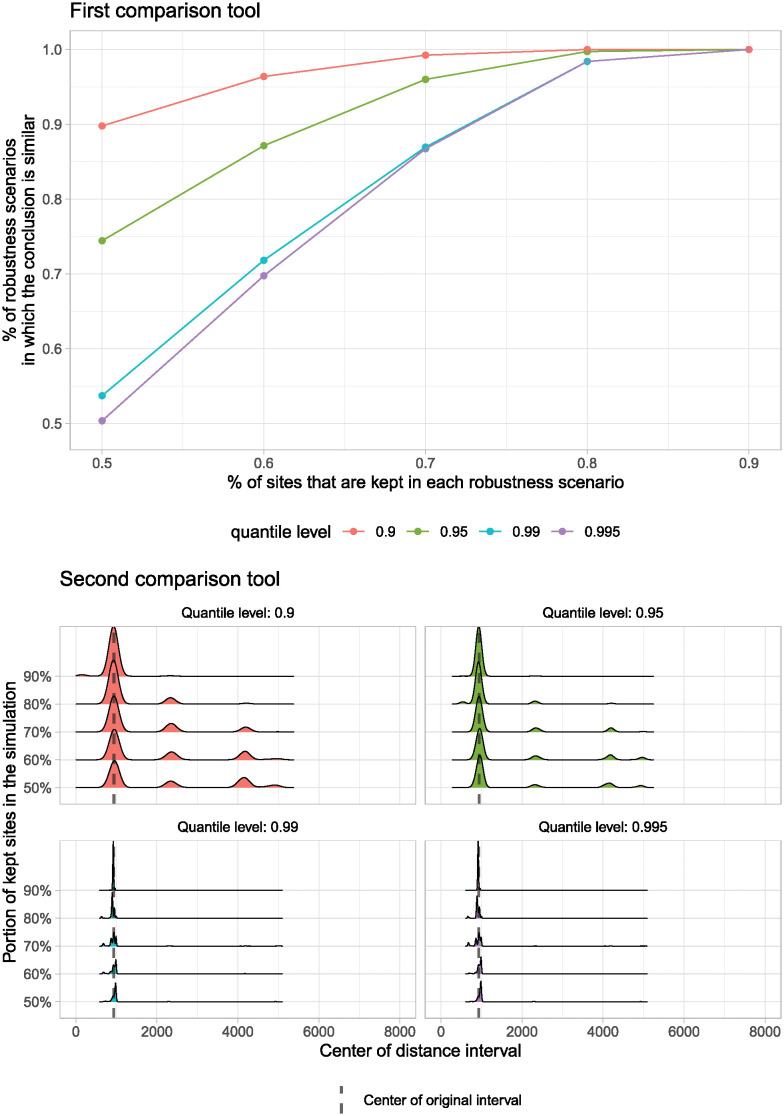
The comparison tools for the second experiment (E2).

The results of the first comparison tool on the weighted random sampling (E2) showed an improvement over the E1 experiment. While at 50% of the data, the results are similar, from 60% of the data onwards, the thresholds for the percentage of robustness scenarios in which the conclusion is similar to the original results are higher. For example, at 70%, there is a threshold between ~85% to ~99%, which means that when doing this type of sampling, which is closer to the reality of fieldwork registries, the 0.995 quantile shows ~15% uncertainty while the 0.9 quantile shows a 1% uncertainty on the model results. For all quantiles, this uncertainty percentage lowered to 0% when 10% of the data was randomly extracted. For both experiments, these results are very promising. However, removing more than 30% of the data increases the uncertainty to levels where comparisons are no longer wise.

Note that the curves obtained in these two plots are smooth and monotonic (increasing). Moreover, they are stable, in the sense that they change very little when the calculations are generated again with a different random seed. These two elements show that the number of robustness scenarios we have selected (n = 10,000) is sufficiently high or, in other words, that the variability across robustness scenarios is controlled.

The second comparison tool provided essential insights concerning the structure of the cluster patterns (Figs 8 bottom and 9 bottom). This tool allows the visualization of the points at which the interval of distances was higher than the CSR quantile, i.e., the density of the center of the interval. As mentioned, we only used the values corresponding to the statistically significant clusters for this analysis. For the E1, it is possible to observe that the clusters get concentrated at similar spatial scales when the highest quantile is considered (0.995). Instead, when the clusters of the various simulations are evaluated against a lower quantile, say the 0.9 one, it is evident that the clusters happen at different spatial distances. For the E2, the results are similar, but as with the first comparison tool, they provide an improved perspective of the patterns. For both experiments, the more sites are removed, the more variability is present in the models. With this tool, it is possible to notice that the clustering happens at different distance levels. Therefore, it is reasonable to conclude that when simulating a large number of scenarios for each of the groups of sampled data, something is happening to the spatial distances at which the clusters are happening. This indicates that there are some levels of uncertainty in the various models. Nonetheless, since the center of the original interval is in all cases located at the mode of the density (i.e. the maximum of the density curve), we can deduce that while there are levels of uncertainty in the data, the comparison tool also allows us to acknowledge that under most circumstances, the spatial clustering is consistent in all the simulations. Simply put, when we remove sites, we can keep our conclusions, and therefore, we are reducing uncertainties in the model results.

## Discussion

### Overview of the analysis

In this article, we looked at the management of uncertainty associated with the application of computational methods, and more specifically PPA in archaeology. We made three contributions. Firstly, we tested the impact of the number of simulations used in calculating the quantile envelope on the result obtained in the archaeological study. We showed that the usual number of simulations used, 1,000, is insufficient because it leads to unstable envelopes. Secondly, we designed a generic framework for estimating robustness. It consists of three building blocks: the observable, the quantity whose deviations we are studying; the experiment, the way in which we simulate the deviations; and the comparison tool, the way in which we compare the deviations with the observed data. By making a choice for each of these, we can fully determine a robustness analysis. Thirdly, we applied this framework to the estimation of the robustness of a clustering analysis of archaeological sites, focusing on the aspect of incompleteness of the sites recorded in the database. We chose the pair correlation function as the observable. Two experiments were defined, corresponding to two probability distributions for sampling the sites to be removed in the random scenarios: uniform sampling and area-proportional sampling. Two comparison tools were used to report the results. On the one hand, we calculated the frequency with which the analysis, carried out on a reduced version of the dataset, leads to the same binary conclusion (there is clustering / there is not clustering) as the original analysis. On the other hand, we looked at the distribution of typical distance levels where clustering was observed (for the scenarios in which there is clustering), in order to go deeper into the archaeological conclusions than the binary of the first comparison tool.

The two experiments provided relevant information regarding the variability in the model results when data was randomly sampled from the original database and used to attempt to reproduce the initial results. The results showed that for the various samples and simulations, when only 1,000 simulations were considered, the Monte Carlo envelope was unstable. In the archaeological literature, there is an implicit assumption that the Monte Carlo simulation alone brings robustness and reduces uncertainties. As shown here, this is not always the case. The comparison tools provided a solution to this challenge. We computed a large number of simulations of the Poisson process of iterations for the Monte Carlo envelope. This provided a more robust scenario to test variability and underlying archaeological uncertainties. However, increasing the number of simulations was insufficient to bring robustness and reduce uncertainties. The second aspect that composed our robust framework for the uncertainties in the analysis was to quantitatively determine the probability for each simulation to contribute to the percentage of robustness scenarios in which the conclusion is similar to the original result. A third and final aspect was our second comparison tool, which allowed the visualization of the recurrence of the cluster patterns in space based on the values of the statistically significant clusters. All analyses indicated that the weighted sampling strategy provided better results than the standard sampling method. This is a relevant indicator that when doing random sampling for computational models, archaeologists should aim to define a sampling strategy closer to the reality of field registry.

## Conclusion

The results from the experiments highlighted the need for a robust framework for spatial analysis and uncertainty quantification in archaeology. When designing a project that uses statistical or mathematical models to analyze non-systematic survey data or legacy data and where going back to the field is not possible, a framework such as the one presented here is a robust option to have a deeper understanding of the spatial pattern. Furthermore, to obtain a more informed assessment of the model results and its variability under a test of uncertainty. The framework presented here contributes to offering clear procedures to explore a database and avoid black-box methodologies, positively affecting the narratives we create about past landscapes.

The result obtained by the comparative tools shed important light on the original study of the Montecristi province. If we extrapolate the results, we can consider that registering up to 30% more sites will not necessarily change the results obtained from the 95 habitation sites. Even when using the higher quantiles for the 50%, the chances of the model being similar to the original data was around 90%. Based on the main author’s years of fieldwork experience in Montecristi and for an area the size of the polygon used in the original study [7], the chances of locating 50 more sites are not very high. What the framework used here has highlighted is that the original model results are accurate and useful for creating interpretations of the spatial patterning, and they can be used for further analysis. Nonetheless, this might not be the case for other datasets in other research contexts.

An important result of the analysis presented in this paper was the systematic revision of the use of the Monte Carlo envelope to test for statistical significance in point pattern analysis in archaeology and Digital Humanities more broadly. While it is known that the envelope will perform optimally with a large database, there have not been studies in archaeology that explore the impact of using Monte Carlo envelopes with small and uncertain databases. This paper provided a robust solution for working with Monte Carlo envelopes and its significant values of the observed site distribution.

A pitfall in applying these methods lies in democratization and reproducibility. While these methods and their interpretation could be feasible for any archaeologist with intermediate computational methods and programming knowledge, they might present a challenge for inexperienced colleagues. Nonetheless, to contribute to the reproducibility of these analyses, the codes for each procedure have been made open access (see Supporting information). Another aspect that might improve this framework and its results is using only simulated data to run the framework. Yet, in this paper, we wanted to test our framework first with real archaeological data.

Overall, we think that this paper can be a contribution to how archaeologists, or any other scientists, think about point pattern analysis as it provides a concrete and reproducible framework for assessing robustness and uncertainties in spatial datasets, particularly databases created in the context of non-systematic regional surveys. The paper offered a reflection on a formalization of what it means to test the robustness of archaeological spatial analysis. We did it by formalizing the process of applying tools to answer archaeological questions, i.e., by answering how many times and in how many scenarios can we get to the same conclusions? And what is the probability for each sample group to reproduce the original results? Finally, this study and its results emphasize the importance of assessing and quantifying potential uncertainties in the data and models, and using transparent and robust frameworks to study archaeological materials. Mindlessly following methods that are supposed to be robust might lead to overseeing uncertainties in the models and assuming they might not have internal variabilities, leading the researcher to write narratives presenting inaccurate interpretations of past patterns and, therefore, creating ambiguous landscapes.
